# Safety of Tiered-Dispatch for 911 Calls for Abdominal Pain

**DOI:** 10.5811/westjem.2019.9.44100

**Published:** 2019-10-17

**Authors:** Tiffany M. Abramson, Stephen Sanko, Saman Kashani, Marc Eckstein

**Affiliations:** * Keck School of Medicine of the University of Southern California, Department of Emergency Medicine, Division of Emergency Medical Services, Los Angeles, California; † Los Angeles Fire Department, Emergency Medical Services Bureau, Los Angeles, California

## Abstract

**Introduction:**

Many dispatch systems send Advanced Life Support (ALS) resources to patients complaining of abdominal pain even though the majority of these incidents require only Basic Life Support (BLS). With increasing 911-call volume, resource utilization has become more important to ensure that ALS resources are available for time-critical emergencies. In 2015, a large, urban fire department implemented an internally developed, tiered-dispatch system. Under this system, patients reporting a chief complaint of abdominal pain received the closest BLS ambulance dispatched alone emergency if located within three miles of the incident. The objective of this study was to determine the safety of BLS-only dispatch to abdominal pain by determining the frequency of time-sensitive events.

**Methods:**

This was a retrospective review of electronic health records of one emergency medical service provider agency from May 2015–2018. Inclusion criteria were a chief complaint of abdominal pain from a first- or second-party caller, age over 15, and the patient was reported to be alert and breathing normally. The primary outcome was the prevalence of time-sensitive events, including cardiopulmonary resuscitation (CPR), defibrillation, or airway management. Secondary outcomes were hypotension (systolic blood pressure < 90 mmHg); or a prehospital 12 lead-electrocardiogram (ECG) demonstrating ST-elevation myocardial infarction (STEMI) criteria or a wide complex arrhythmia. Descriptive statistics were used.

**Results:**

During the study period, there were 1,220,820 EMS incidents, of which 33,267 (2.72%) met inclusion criteria. The mean age was 49.9 years (range 16–111, standard deviation [SD] 19.6); 14,556 patients (56.2%) were female. Time-sensitive events occurred in seven cases (0.021%), mean age was 75.3 years (range 30–86, SD18.7); 85.7% were female. Airway management was required in seven cases (0.021%), CPR in six cases (0.018%), and defibrillation in one case (0.003%). Two of the seven (28.6%) cases involved dispatch protocol deviations. Hypotension was present in 240 (0.72%) cases; six (0.018%) cases had 12-lead ECGs meeting STEMI criteria; and no cases demonstrated wide complex arrhythmia.

**Conclusion:**

Among adult 911 patients with a dispatch chief complaint of abdominal pain, time-sensitive events were exceedingly rare. Dispatching a BLS ambulance alone appears to be safe.

## INTRODUCTION

In the 1970s, priority emergency medical services (EMS) dispatch systems were introduced to help triage 911 calls and resources. Since then, multiple versions of dispatch triage, including criteria-based dispatch, medical priority dispatch systems, and locally developed protocols have been used. However, most dispatch systems have a high rate of overtriage, leading to increased costs, increased utilization of limited resources, and increased use of lights and sirens, all without clear evidence of outcomes that suggest improved patient care.^1^ Many studies suggest that priority dispatch systems lead to overtriage of Advanced Life Support (ALS) units with <1% of low-acuity calls requiring ALS resources.^1^–^6^ For this reason, multiple large cities with accelerating EMS call volumes are re-evaluating their current dispatch systems.

Multiple studies have attempted to identify low-acuity chief complaints and triage criteria at the 911-dispatch level to better optimize allocation of resources.^6^,^7^ Although abdominal pain is one of the most common reasons 911 is activated, few studies have specifically examined dispatch protocols for abdominal pain. The few studies that have been published suggest overtriage and overutilization of ALS resources for abdominal pain with a range from 10–51%.^8^,^9^ Other retrospective reviews found that 84–98% of abdominal pain calls are low acuity and that less than 6–8% were considered true emergencies.^4^,^9^,^10^ Of note, most ALS care was pulse oximetry and/or an intravenous (IV) placement, and when the analysis was restricted to IV fluid bolus, medication, intubation or defibrillation, the majority (19/28) received ALS <10% of time.^7^

Although more than 85% of 911 incidents for abdominal pain require only Basic Life Support (BLS) transport to the emergency department (ED),^8^ many dispatch systems continue to send ALS resources, sometimes in addition to the closest first responder units. In 2015, the Los Angeles Fire Department (LAFD) implemented an internally developed tiered dispatch system (LA-TDS). Under LA-TDS, patients reporting a chief complaint of abdominal pain received the closest BLS ambulance dispatched alone emergency (ie, with lights and sirens) if located within three miles of the incident. If no BLS ambulance was available within three miles, then a closer paramedic ambulance was dispatched, and if no ambulance was available within three miles, a BLS fire company responded emergency along with the closest ambulance non-emergency.

The purpose of this study was to evaluate the safety of this dispatch algorithm by determining the prevalence of 911 patients with abdominal pain and a documented time-sensitive event.

## METHODS

### Setting

The LAFD is a tiered, fire-based EMS provider system, and it is the sole provider of 911-EMS response for the City of Los Angeles. The department covers 480 square miles and serves a population of 4.2 million people. All 911-call takers are sworn members of the LAFD and are either firefighter/paramedics or firefighter/emergency medicine technicians (EMT) who are certified as emergency medical dispatchers. A resource is dispatched to all calls, and there is mandatory offer of ambulance transport to an ED.

LAFD-TDS is a homegrown dispatch system that was implemented in 2015 with the goal of improving call processing times, cardiac arrest recognition, resource availability and response times. Under LAFD-TDS, patients reporting a chief complaint of abdominal pain receive the closest BLS ambulance dispatched alone emergency (ie, with lights and sirens) if located within three miles of the incident. While the dispatch protocol calls for a BLS ambulance, the dispatch protocol dictates that an ALS ambulance responds if no BLS ambulances are available within three miles. Of note, in this system, only ALS providers can perform prehospital electrocardiograms (ECG). However, given that ALS providers may be dispatched to these calls, ECGs are occasionally performed on patients with non-traumatic abdominal pain who met our study inclusion criteria.

**Population Health Research Capsule**

What do we already know about this issue?

*Abdominal pain is one of the most common reasons 911 is activated. While most of these calls are low acuity, ALS resources are commonly dispatched*.

What was the research question?

What is the prevalence of time-sensitive events in patients who call 911 for abdominal pain? Is it safe to send a BLS ambulance alone?

What was the major finding of the study?

*Time-sensitive events were rare (0.021%). Dispatching a BLS ambulance alone appears safe*.

How does this improve population health?

*By demonstrating that BLS alone appears safe, alternative dispatch protocols may be implemented, reserving limited ALS resources for true, time-sensitive emergencies*.

### Study Design

This was a retrospective review of electronic health records for 911 incidents dispatched as non-traumatic abdominal pain from May 2015–May 2018. Cases were included if the patient’s chief complaint was abdominal pain, the patient was the caller or was in close proximity to the caller (ie, a first- or second-party call), the patient was over age 15, and the patient was awake and breathing normally. All calls that met this inclusion criteria regardless of resource dispatched or transport to an ED were included in the study.

The primary outcome was the prevalence of documented, time-sensitive prehospital events that require emergent life-saving interventions, defined as cardiopulmonary resuscitation (CPR), defibrillation, or airway management (including use of bag-valve-mask, supraglottic airway, or endotracheal intubation in a non-ventilator dependent patient). Secondary outcomes were incidents that could potentially benefit from ALS resources and included the presence of hypotension (defined as initial systolic blood pressure < 90 millimeters of mercury [mmHg]) or a prehospital 12-lead ECG that was read as ST-elevated myocardial infarction (STEMI) or wide complex arrhythmia by computer software. ECGs that were marked as STEMI or wide complex arrhythmia were reviewed and interpreted by the authors (TA, ME). Descriptive statistics are presented, including frequencies. We excluded all incidents that were the result of trauma.

Audios from the 911 calls for cases involving CPR, defibrillation, or airway management were reviewed. We used qualitative analysis to identify any themes or key words in the calls. Additionally, dispatch protocol adherence was evaluated. This study was approved by the institutional review board of the University of Southern California (HS-18-00649).

## RESULTS

**Figure 1:**
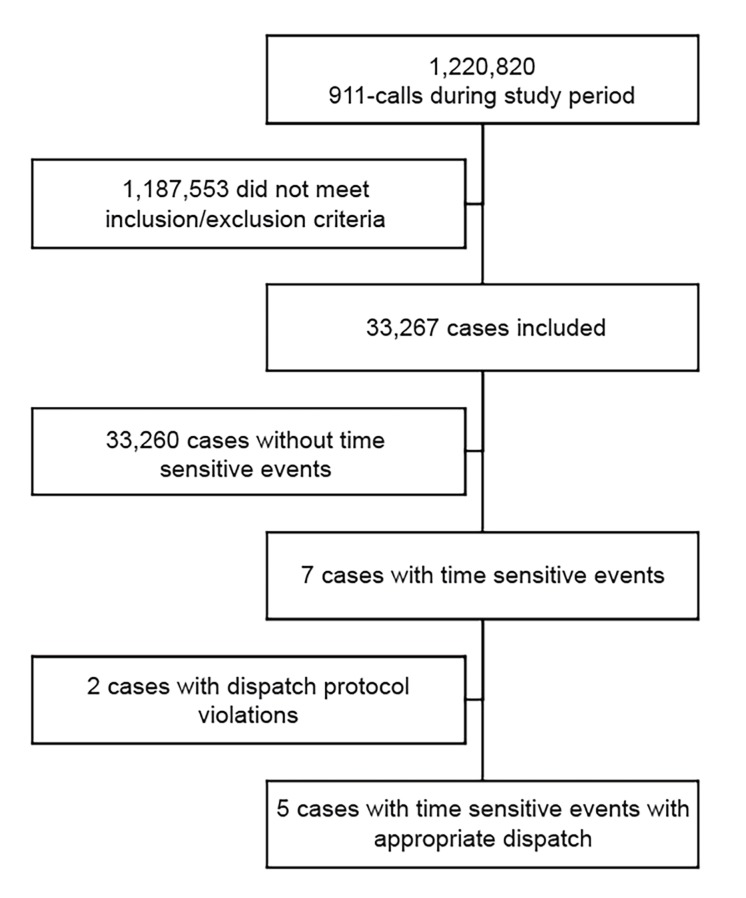
Study flow chart.

During the study period, there were 1,220,820 EMS incidents. Of all incidents 9,999 (0.82%) met this study’s definition of time-sensitive events. Study inclusion criteria was met by 33,267 (2.72%) incidents (Figure 1).

Of the cases that met study inclusion criteria, the mean age was 49.9 years (range 16–111, standard deviation [SD] 19.6) with 7,281 (21.9%) over the age of 65 years; 14,556 patients (43.8%) were male. The mean response time for all included cases was 7.05 minutes (median 6.55, SD 11.52). A BLS ambulance responded alone to 24,248 (72.9%) of the included cases with a mean response time of eight minutes (median 7.43, SD 2.43). In 9,019 (27.1%) calls, a BLS ambulance was not the initial resource dispatched to the scene due to not being available within three miles of the incident. In these cases, a paramedic-staffed engine and/or ALS ambulance were first on scene, and the mean response time was 7.66 minutes (median 7.08, SD 2.32). Transport times were also similar among these groups with BLS-only responses having a mean transport time of 10.34 minutes (median 9.76, SD 3.73) and non-BLS responses having a mean transport time of 9.42 minutes (median 8, SD 3.42).

### Primary outcome

Time-sensitive events were documented in seven patients (0.021%), with a mean age of 75.3 years (range 30–86, SD 18.7), of whom six (85.7%) were over age 65, and 85.7% were female. For calls with time-sensitive events, the mean response time was 6.93 minutes (median 5.52, SD 4.05). Cardiopulmonary resuscitation was required in six cases (0.018%), defibrillation in one case (0.003%), and airway management in seven cases (0.021%). In patients requiring time-critical interventions, including CPR or airway management, the mean age was 75.3 (range 30–86, SD 18.7). Of note, the 30-year-old patient was an outlier who had cancer and was on hospice. Characteristics of each outcome were further analyzed (Table 1).

We reviewed dispatch audios from the seven 911 calls where time-sensitive event occurred. All were made by second-party callers. In two cases, the dispatch algorithm was not adhered to since the callers described the patient as having irregular breathing, which should have prompted an emergent ALS dispatch. Other phrases during the calls that indicated the severity of the patient’s conditions included mention of skin pallor (1); excruciating or terrible pain (2); difficulty or abnormal breathing (2); and mention of chronic medical conditions (2). Details of these calls are included in Table 2.

### Secondary Outcomes

Hypotension, defined as systolic blood pressure less than 90 mmHg, was present on arrival in 0.72% of all included calls. The average age of those with hypotension was 57.4 years (range 16–96, SD 20.6), and 64.2% were female. The mean response time for patients who had documented hypotension was 6.9 minutes (median 6.5, SD 3.2).

A 12-lead ECG was obtained in 2,213 (6.7%) abdominal pain dispatches. In six cases (0.018%), the ECGs met STEMI criteria according to the cardiac monitor software algorithm. Patients with ECGs that met STEMI criteria had a mean age of 61.67 years, and 83.3% were female. There were no cases of wide complex tachycardia captured on 12-lead EKGs. Only three (50%) of six ECGS that were documented as STEMI actually met STEMI criteria when manually reviewed. The inter-rater relatability of reviewers was 1.0. The mean response times for this group was 8.94 minutes (median 6.5, SD 3.2).

## DISCUSSION

Abdominal pain is a common medical reason for 911 activation. In an environment with limited resources and increasing 911-call volumes, minimizing overtriage is essential to ensure ALS resources are available for true, time-critical emergencies. By introducing a tiered-dispatch system that dispatches a BLS ambulance alone for non-traumatic abdominal pain in patients who are awake and breathing normally, there is a potential opportunity to free up more ALS and first-responder resources to respond to true, time-critical calls.

Time-sensitive events were identified in only 0.021% of all cases meeting inclusion criteria, which is considerably lower than LAFD’s overall rate of 0.82% for time-sensitive events for all EMS 911 calls during the study period. The need for airway management or CPR was extraordinarily rare among the 33,000 abdominal pain dispatches under study. Furthermore, in two of the seven cases, if dispatch protocol had been followed correctly, ALS resources would have been deployed, decreasing the frequency from 0.021% to 0.015%, ie, 1.5 in 10,000 patient dispatches. This underscores the importance of a robust, dispatch quality improvement program. Close monitoring, feedback, and education are necessary to ensure that the system is being properly used to protect the public and allow for effective and efficient dispatch protocols.

Hypotension was the most common outcome of interest that was documented. However, it is difficult to infer the clinical significance of these numbers and whether a closer (BLS) first responder or an ALS response with IV fluids would have been of benefit. ECGs that met STEMI criteria were also very uncommon events in this cohort. None of the patients with ECGs that met STEMI criteria were hypotensive upon EMS arrival nor did they require CPR, airway management, or defibrillation prior to ED arrival. Furthermore, 50% of them were deemed to be false positives by the software algorithm.

Finally, there is an association between age and time-sensitive outcomes. Patients who had time-sensitive events tended to be older (mean of 75.3 years old vs 49.9 years old) and female (85.7% vs 56.3%). Additionally, patients with ECGs that met STEMI criteria also tended to be older (61.7 vs 49.9). While patients over the age of 65 accounted for 21.9% of all included calls, they made up 85.7% of time-sensitive events.

## LIMITATIONS

This was a retrospective study of existing electronic health records and possesses the limitations inherent to retrospective reviews, including issues of omitted and incorrectly entered data. However, given the large dataset, we believe this effect to be small. A second limitation is that hospital outcome data was not available for these cases. However, our definition of a time-sensitive event clearly met the threshold of a life-threatening problem. Further studies are needed to analyze characteristics of patients with time-sensitive events, prehospital interventions, and ultimate patient outcomes.

## CONCLUSION

Among adult 911 patients with a chief complaint of non-traumatic abdominal pain, time-sensitive events were exceedingly rare and occurred more often in the female and elderly. In a system with low response times, dispatching a BLS ambulance alone without a closer first responder or ALS resource appears to be safe.

## Figures and Tables

**Table 1:** Characteristics of time-sensitive events.

Event	Proportion of time sensitive cases (n=7)	Mean age (years)	Median age (years)	Over age 65 (%)	Sex (% female)	Dispatch protocol adherance (%)
CPR	6/7	73.5(30–86)	81.5	83.3%	83.3%	83.3%
Airway	7/7	75.3 (30–86)	83	85.7%	85.7%	71.4%
Defibrillation	1/7	77	77	100%	100%	100%
All		75.3 (30–86)	83	85.7%	85.7%	71.4%

*CPR*, cardiopulmonary resuscitation.

**Table 2:** Dispatch evaluation of time-sensitive events.

Case #	Time Sensitive Events (s)	Age (Years)	Sex	Dispatch protocol compliance (y/n)	Dispatch Audio
1	Airway	86	Female	No	“her breathing,” “I don’t think she is conscious”
2	Airway, CPR	85	Female	yes	"my mom needs to go to the hospital," "she has cancer," "she's in a lot of pain"
3	Airway, CPR	30	Female	No	“her face is getting all pale,” “breathing hard”
4	Airway, CPR	86	Female	Yes	“excruciating pain”
5	Airway, CPR	83	Female	Yes	“been in bed for over one month”
6	Airway, CPR	80	Male	Yes	“my husband is very sick” “all of the sudden he has terrible pain”
7	Airway, CPR, Defibrillation	77	Female	Yes	“clammy and weak”

*CPR*, cardiopulmonary resuscitation.
